# Alimentation among Savage and Civilized Peoples

**Published:** 1883-08

**Authors:** Paul Caseneuve


### THE DENTAL REGISTER.

VOL. XXXVII.] AUGUST, 1883. [No. 8.

### COMMUNICATIONS.

### Alimentation Among Savage and Civilized Peoples.

Read before the "Societe Antropologique de Lyon."

BY DR. PAUL CASENEUVE.

[Translated by THOMAS C. MINOR, M.D., Cincinnati, Ohio.]

I do not intend, in the present essay, to enumerate the different alimentary substances used in the past and present among the various races inhabiting the globe—such an enumeration would be too lengthy, and, while it might attract the attention of the curious, could be of no real scientific value—you would leave the lecture-room convinced that French cookery merits the palm above all others, besides, you have already firmly seated convictions on this point.

When discussing alimentation among civilized peoples, I shall present no statistics—my lecture will not treat of social economy. What I propose is a study of comparative physiology, the difference between man in a savage and in a civilized condition, comparing the prehistoric with the modern man, taking for the subject of our meditation a fundamental function of the living organism, i.e.—*nutrition*.

The causes which regulated the nature of man's food during the different ages, causes of a physiological order, of physical and climatic order, causes having their origin in religious sentiment, will all be particularly presented. The sociological consequences of alimentation, the influence of the progress made by cookery on the formation of the family, the appearance of the domestic fireside and the development of the home circle will likewise merit attention. We might even go beyond this, for, alimentation, viewed from a physical and moral standpoint, exerts considerable influence on the organism. No individual can escape this influence. There is an incontestible relation between the character of a people and their food. This is not a conception *a priori;* it is the result of a close scientific course of observations. Let us now proceed to study alimentation in its philosophical and synthetical aspects; in this way we hope to make anthropology instructive and useful.

I.

Alimentation--the action of self nourishing—is a physiological necessity for all living beings, whether animal or vegetable, whether constituted of a single anatomical element, as for instance, a cell more or less perfectly modeled like a monad, or composed of myriads of cells and fibres grouped in tissues and organs like a man. Such living beings are the seat of an incessant movement of composition and decomposition, of assimilation and disassimilation. Life betrays nothing but this intimate collection of workings.

The living machine needs, like the steam engines of modern industry, material to sustain its forces, a reparative alimentation. But while we feed an ordinary machine with raw coal of a simple chemical composition, we cannot nourish any plant or animal save on more complex chemical substances. The plant draws its supply of carbon in carbonic acid, a much more complex body than coal, while animals need substances still more complicated.

Our food, aside from simple animal substances, chloride of sodium, phosphates, etc., and water, is composed of matter called albuminoid, more or less analogous to the white of an egg; amylaceous matter, as in fecula or starch; fatty matters, as oils or fats. These latter substances, termed organic, are very complex as regards chemical constitution, and, remarkable to state, are produced by living beings themselves. Vegetables especially, produce starchy and fatty matters, while animals especially produce albuminoid and fatty matters. What happens then? The plants serve to nourish many animals, and the animals in turn become the prey of those stronger beings who sacrifice them.

A savage man pressed by hunger, or famished, instinctively eats all the plants and animals he comes across. He is polyphagious. He eats when he can, that which he can, and where he can. The sense of taste developed among civilized men is only a rudimentary sense among savages.

Native Australians eat lizards, ants, and snakes, and consume the roots of vegetables, among others the root of a species of fern which they first bruise between two stones. They chase the kangaroo, which they devour with avidity, hardly taking pains to broil the meat externally.

The Andamanites, who inhabit a small archipelago on the Gulf of Bengal, live on turtles, oysters, and raw fish which are often putrefied; they also love the meat of wild cats and wild hogs.

Naturally, I am now presenting the lower types of human beings, who neither lead a pastoral nor agricultural life, but live as hunters and fishers.

The Fuegans of Terra-del-Fuego are most miserable. A few varieties of shell fish, a vegetable parasite, a species of mushroom growing on beech trees, which abound in that country, and a few herbs compose the nourishment of these savages. When a whale is washed ashore in a state of putrefaction these poor creatures enjoy a regal repast. If they catch a live fish or bird it is eaten raw, while yet living. When famished they are cannibals, and eat all their old women, preserving the lives of their dogs which aid them in the chase. In other South American regions, the savages suppress useless mouths—they strangle the aged and bury them.

The Bushmen, Caffirs, and Hottentots of South Africa eat insects, larva, and putrefied meat.

The natives of Manyonema, near Lake Tanganyka in Central Africa, practice anthropophagy from necessity and have no repugnance to disinterring the remains of relatives who have died from disease, often, even before eating, permitting the remains to still further putrefy by exposing the body in a current of running water. These Africans instinctively eat all the carrion they come across.

Our refinement is shocked by merely reading the statements of travelers worthy of belief. To eat putrefied whale and rotten human bodies seems the very acme of savage bestiality, a vile and horrible appetite which places man on a level with savage carnivora. In the meanwhile if we study the substances consumed by people claiming to be civilized, we find the most depraved tastes, tastes which will serve to shock those of our descendants who in the future will likewise study their ancestors. We do not eat putrefied whale to be sure, but we do eat snipe and pheasant in an advanced condition of decomposition. Where is the connoisseur sportsman, who has not wrinkled his eyebrows in sorrow, on seeing a snipe defecate before the fatal bullet has struck its mark? And if, the housewife, more civilized than her master, empties the snipe's entrails, what a storm of wrath is unloosed: If the bird is not served up *bloody*, the devil is to pay.

And cheese; Great Jupiter Ammon!

"A dessert without rotten cheese is like a beauty who has lost one eye," says Brillat Savarin.

The illustrious gastronomist, of Bugey, speaks, let it be well understood, of *putrefied* cheese. Assuredly there are all degrees of putrefaction in cheese, but, in some *creameries* the caseine is allowed to decompose to the last degree, the rancid fatty matter giving off the lively stink of butyric acid. Certain lords and ladies accomodate themselves very well to this kind of barbarous nourishment. I may go further and say, civilized epicures consider it a great dainty.

Are not these comparisons curious and very interesting? Do they not show that, whatever may be the distance separating us from the savage man, we shall meet certain habits and certain tastes which are similar? On more than one occasion before this society we have shown the similarities existing between widely incongruous peoples, the one the outcome of a progressive evolution, the other the victims of a barbarity without remedy. All races have a common point of contact.

The savage, as we have before stated, eats when he can--when there is no nourishment he fasts. To cheat hunger, like the New Caledonian* of the Loyalty Isles, he at times eats white clay charged with organic detritus gathered from the clefts among the rocks. He thus fills his stomach and relieves his hunger.

* Our author need not have gone so far for specimens of the dirt eater. In our own glorious Republic, the clay eater is still plentiful in the Carolinas.—Translator.

If the New Caledonian meets with an abundance of food, a rotten whale, for instance, washed ashore by the kindly waves, his joy is intense and is only exceeded by the fulness of his stomach, for he can swallow an enormous amount of such putrefied meat, ten to twelve pounds being ingested at a single meal.

Captain Gray, quoted by Lubbock in his remarkable work entitled "*L'Homme avant e'histoire*," gives a graphic description of the unfortunate Australians dancing and crying for joy when a rotten whale was washed ashore. "They rubbed its blubber all over their naked bodies and made their favorite wives do the same; after which they opened a passage through the whale's fat down to the lean meat, which they ate sometimes raw, sometimes broiled on the end of a pointed stick. As other natives arrived on the festive scene, their jaws vigorously worked on the carcass of the whale, and you could see them climbing over the stinking mass in search of the more delicate morsels, showing there are even epicures among New Caledonians. For entire days, the natives remained near the carcass, covered with fetid fat from head to foot, gorged with rotten food to satiety, engaged in continual quarrels and wrangles, affected by a disgusting kind of skin disease produced by this horrible food, and presenting a disgusting spectacle which defies description."

This gluttony which is common among savage peoples is, at times, seen among civilized peoples.

What parties deceive themselves like Harpagon, and write over their dining-room doors not: *It is necessary to eat in order to live*; but, "*It is necessary to live in order to eat.*" I not only allude to the orgies of the highly civilized Romans, but to those stomachs at the present epoch which enjoy revolting repasts.

We might cite the quantities of beer drank to gain wagers, which exceed any stories that the imagination can invent, without even alluding to the solid food consumed.

In the midst of the most civilized peoples, live beings who seem to be the direct social scum of the Papians of Tasmania. My friend, Dr. Lacassagne, recently compared the criminal man, the savage man, and the primitive man. Similarities, from a moral and intellectual standpoint are as true as from a physical standpoint.

If we analyze the passions of inferior beings in modern society, we shall find the living witnesses of the natural law of evolution, appear across the ages like stigmas on our poor humanity, leading man to forget his modest origin and his feeble nature.

### II.

Like the savage of the present day, the prehistoric man submitted to the necessities of his circumstances and surroundings. Frugivorous and vegetarian, perhaps from instinct, he was undoubtedly carnivorous when the occasion demanded. The remains of his food are still found at prehistoric stations; we may cite in passing two celebrated examples, the grotto of Aurignos, the station of Solutre,—consisting of the skeleton *debris* of animals belonging to species existing at a prehistoric period. We meet the bones of the bear, mammoth, rhinoceros, the ures, ure-ox, and primitive bull. The gigantic wood elk and the reindeer are equally represented. Later appear, the wild goat, the sheep, domestic goat, the wild boar, domestic hog, the dog, and the fox.

We find particularly the long bones of the animals broken. Surely it was the marrow in the interior of these bones that was sought by the primitive man even as by the savage man of the present day. The Andamans always break the bones of the hog in order to eat the marrow.

We also find the remains of the prehistoric kitchen on the coast of Denmark, designated by the savants of that country as *Kjoekkenmoeddings*; this *debris* which, has been preserved since thousands and thousands of years before our era, gives an idea of the nourishment afforded the prehistoric man. There were mollusks, bones of mammalia, birds and fishes. We might cite oysters, mussels, cockles, periwinkles, brown bear, dog, fox, cat, lynx, martin, otter, seal, porpoise, beaver, water rat, mouse, wild swan, black grouse, penguin, herring, cod fish, mud fish, and eel.

Some of these animals belong to the extinct fauna, some have almost disappeared from Denmark, or have diminished in size. The black grouse, for instance, has been crowded back to the pine forests of Northern Scandinavia. Denmark deprived of its forests could no longer sustain it. The oyster has not disappeared, but it is smaller than in former times, if we are to judge from the largest specimens found.

The discoveries of these *Kjoekkenmoeddings* in Denmark has been followed by very numerous discoveries of a similar nature at various points in France, Belgium, Australia, and America. We do not meet human bones as in the caverns belonging to a more primitive period, nor do we find those grains and cereals, which prove that the kitchen *debris* belonged to an agricultural people.

The agricultural epoch seems to agree with the period of the lacustral cities. The discovery of loaves of bread and numerous cereal grains clears up all doubt on this score, and, in this connection, we may remark that these grains were bruised and broken up in a mortar by means of a round stone. To-day, the savages of Africa employ the *merhaka*, or similar stone mortar. This primitive method of crushing substances is still employed at the present time on the continents of Europe and America, and the porcelain pestle and mortar of the apothecary have been the seal of remote antiquity.

As regards alimentation of the prehestoric man, we find in his kitchen *debris* properly speaking, coal cinders, pieces of pottery, and instruments made of silex or deer horns. The art of cooking food was then known at the epoch when man dwelt in caves.

To what period of time must we go back in order to discover the commencement of that cookery which contributed to make mankind civilized? That period is lost in the midst of by-gone ages and was contemporaneous with the discovery of fire.

From the day that the prehistoric savage, endowed with an elimentary sense of observation, discovered that two pieces of dry wood rubbed against each other would develop heat—he patiently pursued the process until the temperature was elevated sufficiently to provoke a flame.

Even in our day we find natives of Australia, Sumatra, Caroline Isles, Kamtschatka, China, South Africa, and America, using a wimble or *fire drill* which consists of a sharp pointed stick of which one extremity is inserted in a cavity hollowed out in another piece of dried wood. These pieces of wood are rapidly turned by both hands, while a powerful vertical pressure is kept up. The instrument has received many modifications which I need not here describe.

Primitive man, like the savage of to-day, exposed his meat directly to the fire, suspending it by pieces of wood or other material. The Patagonians still cook their meat between two heated stones; they likewise warm water by throwing hot stones in their water vessels. Virchow has noted a remarkable instance of similar kind in Germany, where among certain people, a red hot iron is plunged into punch in order to make the liquor hot. It is thus we perpetuate across the ages those little customs whose orgin we are ignorant of unless we have studied the customs of the past. This was not the only way in which water was heated, for it was placed on the fire in a large clay vessel often unbaked. Assuredly such pottery was not impermeable, but it sufficed to contain the larger portion of the liquid. The Micamac Indians, of Nova Scotia, according to Hart, made vessels destained to heat water. These vessels were made from the bark of certain birch trees (*petula papyracca*). The Scythians, according to Herodotus, boiled the flesh of animals in vessels made of skin. A pot, no matter what its shape or size may be, is a much more civilized utensil. The baking of clay and the polish given to such pottery by the use of lead salts shows what modern industrial progress has achieved.

Many tribes at the present day still make hand molded pottery, the same that their ancestors made in prehistoric times, after their hyena period had passed.

The commencement of family life dates from this epoch. The woman guarded the fire, which, difficnlt to produce, had to be kept up, and woman, being mistress of the fire and the kitchen, made, as she still makes to-day among savage peoples, all the pottery. Man, in the meantime, pursued the chase and fishing, and furnished the family food. This mutual dependence existing between man and woman was created by the desire for nourishment, and was the starting point of family life, securing bonds of domestic affection as closely as the ties binding modern households together.

### III.

What distance separates our civilization from that remote period when the art of cooking first originated? I will not speak of condiments and seasonings which vary to infinity, but of the simple method of cooking which gives such savors that only a refined palate can appreciate them. A meat broiled on a gridiron, baked in a close stove, stewed in a pan, or basted on a spit, gives off, following the method of cooking adopted, a special odor which is never mistaken by epicures. I will state in addition, that the same kind of meat cooked on a spit by different cooks will present different qualities. To cook a roast of beef well requires great experience—and we are led to believe in the aphorism, "One may become a cook, but one must be born a roaster." Throwing aside the exaggeration of this proposition, it is nevertheless true that cooking is a difficult art, never acquired by the unintelligent, and requires a true spirit of observation.

Let us add that we have perfected drinking like eating. Water drinkers in France are rare. Primitive man, like the savage of our day, went like an animal to the sources offered by nature; at other times he tasted the water in the succulent fruit of the summer season. But one day he put aside some of these ripe fruits for future use. Alcoholic fermentation, that phenomenon produced so rapidly in sugary juices, was developed. The discovery of fermented drink was made. The intoxicating and exciting properties of these drinks, which carry our souls to the elysian fields of happy dreams and sweet illusions, and, which, for the moment, chase away and dispel earth's heartaches and moral sufferings, were well known and highly prized among primitive races. Even at the present day almost all savage races use fermented drinks, the New Caledonians alone, it appears, being ignorant of drink and the drunkenness it procures. This is the one singular exception.

Among civilized peoples, the consumption of these drinks, particularly alcohol, which is really the exciting and intoxicating principle, has assumed such proportions that sanitarians have become alarmed. It has even been designated by the name of

*alcoholism*, the morbid condition resulting from the abuse of these drinks.

The discovery of fermented drinks dates back to very ancient times—assuredly it is difficult to meet vestiges of these products but we can suspect from the remains of the fruits found that they may have served in making drinks. Dr. Lioy discovered dog berry fruit in Lake Fimon, and has been asked whether this fruit was not formerly used in the manufacture of drinks. On his part, M. Gabriel, of Mortillet, thinks that ripe raspberries and blackberries were likewise used. On the other hand, if it is true that grapes are found in the swamps of Parmesas, the knowledge of the vine goes back to the most romote prehistoric period of time.

The fermented drinks consumed by the savage of the present day, by the peoples of antiquity, and by the modern civilized peoples are innnmerable. This large variety of drinks is explained, if we reflect that all sugar and starchy products are easily fermented and produce alcohol—sugary products directly, starch products after the change of starch to sugar. Every race of peoples, following the nature of the products furnished by its soil, manufactures that drink that experience has taught it to make.

Hereditary habits have become intermixed, and nations of today have finished by adopting for use certain preferred drinks. The quality of these drinks varies with latitude. In the meantime it is hardly necessary to remark that the free commercial communication that now exists between civilized nations has to a large extent done away with the making of certain exclusive beverages formerly used, although savage and semi-civilized peoples still to a large extent cling to their ancient drink.

Starchy materials serving for the manufacture of fermented liquors are usually derived from the cereals, such as wheat, barley, rice, and corn. Sugary substances furnished by vegetables and animals are also employed, as for instance milk, honey and all kinds of fermented plants.

The *Zythus*, of the ancient Egyptians, the *Booza*, of modern Egypt, the *cerevesia* of the Germans, and the *beers*, are made from cereals. Beer is made from germinated barley. Under the influence of this germination the starchy or feculent material is turned into sugar. The germinated barley once boiled gives the must which afterwards ferments.

The saccharifying action of germination is a scientific process indicating a gift for observation among an already cultivated people, but savages, thanks to a more elementary observation, attained the same result. They recognized that in chewing corn or rice, and gathering the product mixed with saliva and allowing the mixture to rest for a time, that an intoxicating drink was produced.

The *Chicha* of South America is made by this disgusting process. In the Isle of Formosa an intoxicating drink is made from rice in the same manner.

We have a scientific reason for this primitive process of production. The starch is saccharified by a ferment contained in the saliva, similar to the ferment contained in germinated barley. This is called the *diastase.*

The Japanese manufacture a very strong beer from rice known as *Sacki.*

In Java, the natives prepare two kinds of fermented liquor, one named *Bodik*, made from boiled rice; the other named *Brom*, made with *Ketan* or glutinous rice. They employ as a ferment *razi* which contains onions, black pepper, and pimento. The *Brom* is burried in the ground for several months, enclosed in tight vessels. This burying process is also used in the manufacture of *Chicha* in South America. When an infant is born the natives make a quantity of this liquor, to which is added a large amount of meat. This drink is not consumed until the child has grown up to manhood, and is then used on the wedding day.

The Manachoux Tartars in the same manner prepare a celebrated liquor known as *lamb-wine*, by mixing lamb's flesh with milk or starch, which is consumed after fermentation sets in. But the most common of all drinks among the Tartar and Mongolian tribes, used since the most remote period of antiquity, is *koumiss* or *kuniz*, which is nothing but fermented milk. The milk contains a special sugar which undergoes alcoholic fermentation like ordinary sugary juices. According to Herodotus, the Scythians used *koumiss* long before the Tartars.

Honey, that saccharine material elaborated by bees, serves to make one of the most ancient of fermented drinks, this is *hydromel*, still made at the present day in Russia, and in Africa among the Hottentots, and by the natives of Madagascar.

As to the fermentable saccharine plants used for making drinks, they are numerous. I will cite the maguey—a species of aloes, which furnishes on fermentation the Mexican with *pulque*. The figs, pomegranates, and other fruits served in Egypt for making artificial wines—as to wine properly speaking—it is a widely used beverage at the present day. The grape, of all saccharine fruits is the most used in making wine. The ancient Hebrews, Egyptians, Assyrians, Persians, Greeks and Romans praised it highly. Among the Arabs the wine of the date palm is held in great esteem.

In France cider and poire, the first named made from apples, and the second from pears, are largely used in the Northwest.

In India, the *asclepias acida*, when fermented gives a sacred drink—the wine of Soma. According to the legend Indra found this treasure in heaven, hidden like a bird's nest in a rock, among the cliffs surrounded by bushes. *Soma* is made in the following manner: "The stems of the asclepias are bruised with stones and the exuded juice collected on a goat-skin filter, this skin is then pressed between the two first fingers, which on this occasion are ornamented with gold rings, afterwards the filtered juice is mixed with barley and clarified butter. When the liquor has fermented, one spoonful is poured out for the Gods, and another for the Priests, the maker of the liquor crying out, "The drunker thou art, the more propitious thine acts." In one of the hymns of the Rig-Veda, Indra is called "Drinker of the wine of Soma, hurler of the arrows of lightning and thunder, dispenser of fecundity to large jawed cattle."

But man, not content with intoxicating drinks, has endeavored to withdraw the active principle of intoxication. With the birth of chemistry, alcohol was extracted by distillation. To-day this alcohol mixed with sugar and aromatics makes the numerous table liquors so commonly used by civilized people.

Experience has also taught mankind that alcohol is not the only principle acting on the nervous system and giving rise to that artificial excitation that leads one to look at life through an enchanted prism. Tea in China and Japan, mate in South America (Paraguary), guarana in Brazil, cocoa in Mexico, the coffee in Arabia, are all widely used and exciting substances. Civilized Europe has derived from these countries of origin tea, coffee, cocoa—articles used for daily consumption. All these owe their special property to a well defined chemical principle—*caffeine*—which acts especially on the nervous centres. Other substances are likewise exciting. *Tobacco* is largely used in Europe, America and the East, *opium* in Asia, *hasheesh* in India; all these substances in small quantities excite the brain, in increased doses they stupefy and throw the organism into a condition of progressive torpor, thus creating a sort of artificial life in which illusions and hallucinations are closely united to reality in a smiling but deceitful mirage.

### IV.

I now approach the really philosophical and interesting portion of my subject, and will treat of the causes which influence alimentation, the psychology of nutritive wants. It offers me a chance to show you that man, like animals, is essentially a plaything in the midst of physical conditions, due to his unconscious hereditary aptitudes. Man acts, we might say, owing to the natural causes leading him on.

Hunger, that imperious mistress, commanded savage man as she did primitive man. All physiological nutritive materials suffice to satisfy those inferior races whose digestive strength, wholly animal, is served by a strongly developed sense of taste. We might correct the aphorism of Brillat Savarin and say, "*Men and beasts feed themselves; civilized man eats; but, only a man of intelligence knows how to eat.*"

When our civilized races are pressed by the same starving wants, you will again discover the savage man, with his gross appetite and barbarous passions. Without going back very far in the first centuries of the present era, we find in the modern history of European nations well authenticated cases of anthropophagy. Schiller reports that at the end of the thirty years war the Saxons had become cannibals. During the famine that desolated France in 1030, they hunted men; a butcher was condemned to be burnt for having sold human flesh on the march to Tournay.

Pierre De e' Estoile, in his chronicles, gives some curious details regarding cannibalism among the Parisians during the siege of Paris by Henry IV, "Good King Henry," in 1590. It was the case of a very wealthy lady, who having seen her two children die of hunger, made her servants salt down the bodies, which were afterwards eaten. Some of the foot soldiers also chased men through the streets of Paris and ate the bodies of these hunted victims at the Hotel St. Denis and the Hotel Palaisian.

I find in the "*Decades of Louis XIII*," by Legrain, that the people dug up the body of Marshall d' Ancre, and that one of them cooked the Marshall's heart over a charcoal fire and ate it, seasoned with vinegar.

There are, no doubt, on the other hand, more refined beings in our civilized epoch who would die of starvation, powerless to vanquish their repugnance, rather than accept such a savage diet, but such refined beings are far from constituting a majority. "Scratch the polish from civilization," says a humorist, "and you will always find the savage beast in its appetites."

The causes which regulate alimentation among civilized people are no more limited than among savages. They are multiplied. For the cultivated man is directed in his choice of food by an exquisite and delicate taste, and is capable of establishing the most subtile distinctions between nutritive materials. To give an idea of this, I need only cite the cases of gourmand epicures, who can distinguish by merely tasting wines the nature of the grape and the year of its vintage.

Besides civilized man has at his disposition the products of soils far remote from his own country. Rapid transit brings him the products of foreign parts. Moreover economical causes intervene in our refined modern society; and accordingly as he is pecuniarily able, man may choose his own food. Owing to the large variety of food placed at our disposal, the most varied tastes are cultivated, undergoing at the same time those modifying influences still badly known as heredity, assuming a really strange character of diversity.

The joint liability of taste and digestion forces this diversity on the digestive faculty of the stomach, and surprising individual peculiarities are noticed. Women, in particular, present these individual varieties in a very striking way. They have an insurmountable repugnance for this and that kind of nourishment, and the stomach revolts like the taste. "*Alas! Indigestions are for polite society,*" said Voltaire. This is true, for it is among cultivated and refined people that we meet those capricious digestive faculties, those susceptible and feeble stomachs. Physicians have characterized such individual aptitudes by the word idiosyncrasy. This theoretical expression as regards savage men, corresponds to the reality of a fact in the case of man civilized.

Imagination will often lead us to compare the idiosyncrasies of unpolished and refined people.

Climate has an incontestible influence on alimentation; but less in the case of the savage than in civilized man. We often read, in works of physiology, that the Esquimaux eat much fat in order to exist in a cold climate. We know, in fact, that food very rich in combustible elements, carbon and hydrogen, forms material for combustion of a high order. I believe this example to be badly chosen. The Esquimaux, like all other uncivilized people, eat what they can obtain, and hunger excels all other influences. Besides if we come to look at the alimentation of savages living in a warmer climate we see that they too are fond of fats. Captain Cook states that the New Zealanders drink oil in a ravenous manner, emptying lamps and even swallowing candles, crowding around the boilers in which he was melting the fat of sea lions, and swallowing grease like covetous children swallow bonbons.

My opinion is, that savages indirectly submit to the influence of climate only following the quality of the nutrition furnished. Climate influences the fauna and flora of a country and the savage naturally supplies his wants with the products he meets, having no choice in the matter. Among civilized people, more sensible as to the action of their surroundings, man can choose his food, and we can more clearly observe the influences exerted by climate. There is no doubt that the population of Central Europe had a different alimentation from that of Northern Europe, and even in the same regions, following the seasons, we adopt by preference, special articles of diet. Excessive heat paralyzes the digestive functions and diminishes the appetite, and condiments and stimulants of all sorts are eagerly sought after. Cold, on the contrary, leads us to adopt all kinds of food without complaint.

Climate and temperature have as great an influence on the quantity of nurishment ingested as upon its quality. It is certain that savage man, like civilized man, in a general way eats more or less, following the physiological necessity of increasing or diminishing the elements of combustion.

I now come to a cause of the moral and psychological order that presents great interest. I desire to speak of the influence of religious sentiment on food. If we analyze this influence we discover that religious sentiment determines the adoption of certain foods in some instances and in other instances rejects them. We see besides the quality of the food, the meal itself, under the domination of religion, becomes a solemn ceremony. Funeral dinners in honor of the dead and of the Gods are examples of this kind. Fetichical ideas in regard to food also lead to the performance of curious acts. We know that the Fetich materializes the qualities of what he consumes and the matter of life thus absorbed is thereafter inherent to the consumer, for in eating any living being they are supposed to become impregnated with its qualities. The Malays, of Singapore, eat tiger meat, not because they love it, but because they believe a man who eats tiger acquires the sagacity as well as the courage of the animal. The Dyaks, of Borneo, have a great prejudice against venison, and only women and children eat it. The men avoiding its use because the deer is a timid animal and its flesh would lesson their courage. The Dakotah Indians eat dog meat in order to acquire the sagacity and courage of the animal. The New Zealanders make their infants swallow clots of blood in order to make them insensible to pity. The Caribs will not eat hogs or turtles for fear their eyes might become small like those of these detested animals. In times of antiquity did they not believe that to eat frogs was to increase fecundity because frogs deposited large numbers of eggs? These Fetichical prejudices are precisely one of the causes of anthropophagy, aside from anthropophagy from necessity, that which I spoke of at the commencement of my lecture. The New Zealander eats his most formidable enemy in order to attain that enemy's ferocity, bravery and cruelty. When a chief was killed the law required that his wives should be given to the conqueror. Then the body was roasted and devoured under the direction of the high priests or *arikis*, who afterwards feasted on choice morsels of the victim. The left eye of the cadavera, in which reside the soul, was especially sought after, and the man fortunate enough to receive this titbit doubled his own spiritual being. At the present day modern society evinces similar sentiments. Lubbock cites a little girl who said to her brother, "If you eat too much goose you will become as silly as the bird." This reflection on the part of a child is singularly instructive and picturesque.

In many places they curiously confounded the victim and the divinity, and adored the first before sacrificing it to be eaten. Thus in ancient Egypt, the cou apis was at the same time God and victim, and some suppose that Iphygenia and Artemis were a single person.

In Mexico, at a certain season of the year, the High Priest of Quetzalcoaltl made an image of a God formed from flour mixed with the warm blood of small children. After a series of imposing ceremonies the priest pierced the image with an arrow, it was then removed to the Palace where the King ate a portion, and the remainder was distributed to the waiting multitude outside.

The great annual sacrifice in honor of Tazcatlipoca was also very remarkable. They chose for a victim a prisoner of war, the handsomest young man they could select. For the space of a year they treated the victim as though he were a God. As he passed along, the population prostrated themselves before him, rendering the homage due a divinity. The last month they waited on him with particular care, giving him all the luxuries the land afforded, as well as all the enjoyments, for four of the most beautiful native girls were wedded to him. Finally, the fatal day arrived, they placed him at the head of a solemn procession which slowly wended its way to the temple. Here the handsome young man was sacrificed with great ceremony, and with all marks of respect, then horrid climax! the priests and chief men of the nation partook of his body.

Doctor Short speaks of a singular sacrifice made by the Khonds of Central India. "They fix a stake very firmly in the soil and bind to it the victim, who remains seated on the ground. After annointing the unfortunate with oils and perfumes and covering him with flowers the assembled tribe worship him all day. In the evening they all indulge in a debauch. The third day, in the morning, they make the victim drink milk, afterwards the High Priest implores the Diety and asks a benediction for the faithful, praying that they may increase and multiply, that their cattle and poultry may thrive, that their fields may be fertile, and in the end that all may be happy. Then the priest recounts the origin of the ceremony which he intends celebrating, telling the people it will certainly shower blessings on them, and concludes by saying that he has obeyed the orders of Diety in thus assembling the chosen people together. Now, by means of chants and prayers, the priest excites the multitude to compassion. After this singular ceremony he seizes the victim and drags him into the sacred grove of trees where the final sacrifice is accomplished. In order to prevent any resistance on the part of the victim his legs and arms are broken and he is stupified with opium and datura, then the Jan or priest strikes the unfortunate on the head with an ax. The crowd immediately rushes forward, each one determined to secure a piece of the sacred meat, and, in a few moments, nothing but the bare white bones rest on the earth."

They eat their God in order to propitiate him. They eat him besides to bear witness to their oaths; thus, in some portions of Africa, "to eat a Fetich" is a solemn ceremony that women perform in order to swear fidelity to their husbands. Men swear fidelity to their friends in the same manner. In the ceremony of marriage at Issim, the betrothed couple "eat a Fetich together as a proof of friendship and as an assurance of fidelity on the woman's part." Modern religions, among civilized people, present certain ceremonies that merit comparison, viewing the matter from a philosophic standpoint, with savage customs. I must hasten to add that barbarity and cruelty are fast disappearing and mystical and purer ceremonies are being substituted.

To the religious anthropophagy is connected anthropophagy from filial piety, which also finds its explanation in Fetichical belief. The Battas, of Sumatra, eat their aged parents after sacrificing them in order to absorb their qualities, and thus in a manner incarnate their virtues. This sacrifice is accompanied by grand ceremonies. Such customs, so very strange and curious, are they not the exaggerated translation of a very human sentiment found in all tender and passionate natures? Who has not seen the loving and gentle mother saying to her babe, which she presses tightly in her arms, "I love you so much that I feel like eating you?" She wishes to revive in herself the being she has nourished at her breast. She devours the babe with kisses; she gently bites its little chubby fingers, and abandons herself to the pleasure of tenderly pinching it, and even then her affective passion seems only half satisfied.

So savages eat their aged parents whom they love, the civilized mother is contented with embracing and caressing her loved ones. This is progress! Old parents, it is needless to remark, do not disprove the more civilized method.

While I analyze the feeling that presides over alimentation in all its forms, I do not wish to omit describing judicial anthropophagy. These same Battas, of Sumatra, practice it. The adulterer, the night thief, and the assassin are condemned to be eaten by the people. The criminal is tied to a post, his limbs separated in the shape of a St. Andrew's cross, and the assistants, at a given signal, pull the unfortunate apart, tearing his quivering flesh into ribbons.

Beside the influences determined by religious sentiment on the nature of alimentations, I wish to describe the inverse prohibitive influence.

The law of the prophet (Koran) forbids a good Mahometan drinking wine or spirits. Greeks and oriental Christians view the drinking of koumiss as a renouncement of faith. The followers of Zoroaster, the ancient Scandinavians, the Britons under Cæsar, would not eat a hare; the modern orthodox Jew never eats pork; the Brahmans and Buddhists never eat meat of any kind. And in a great modern religion, the fasters, the Friday abstainers from meat, also reject certain alimentary substances.

I can only describe in passing those interesting customs found in all religions, and which at times owed their origin to hygienic considerations that had not escaped the notice of the great founders of those religions. The abuse of alcohol and the unhealthiness of wormy meat often constituted the cause of this proscription. I likewise hesitate to persue my ethnographical studies across the ages, and thus describe to you the various ceremonies once so commonly enacted. How in antiquity, funeral repasts were served in honor of the dead, how public repasts were given to conciliate the Gods. You will find in the "*Cite Antique*," of Fustal de Coulanges, that eminently classical work, long recitals of such customs intensely interesting and worthy of serious thought.

V.

There remains one chapter of the highest physiological importance that I can only lightly touch on. It treats of the influence exerted by food. Animals, like plants, are strikingly affected by these influences. The culture of plants rests on a well known alimentation which often produces varieties exceptionally constituted. The art of raising animals, permits us to obtain, by using special foods, astonishing results of variety and beauty. Form, height, color, may thus be modified. Man, moreover, does not escape this influence, and this remark is applicable, not only to man's physical development but also to his intellectual capacity. Our intellectual work, the activity of mind and our inspiration are all different, following the liquids we drink, water, wine, tea, or coffee. Our humor, desires, feelings, vary according as we are hungry or surfeited.

Individual collectivities likewise undergo this influence. The national character of the English is not to be compared with that of the Irish. The latter live on potatoes. The English live on meats. It seems that the English find in their alimentation, rich in nitrogen, an intellectual strength that is sadly lacking in their tributaries. The Chinese eat rice, like the negro race, and seem powerless to accomplish those efforts that often decide the fate or destiny of a people.

Most assuredly I do not pretend to connect the future of any people with its alimentation, but it is none the less true that food is a factor which must not be overlooked by the philosopher who investigates sociological laws. These laws are multiple; the more important, as the more secondary meriting mention.

I here terminate these considerations regarding alimentation among savage and civilized people. Every fable carries with it a moral. Every scientific comparison must have its conclusions. Many have desired to widen the gulf between the human and animal kingdoms. The facts we have reported are sufficient to show how artificial this gulf is. We have seen, in fact, by studying one of the fundamental functions of the organism, nutrition, that savage or primitive man was closely allied to the beast. A wider distance separates civilized man from the savage, viewing the subject from an intellectual standpoint, than separated savage man from anthropoid apes.

On the other hand the perfect man has not yet appeared upon the earth. The primitive man was an inferior man—a true animal. The relative perfection determined among civilized races is only the fruit of a long evolution of progress.

These scientific truths may be opposed by certain received beliefs. The scientific man need not be troubled by the consequences of his discoveries. His only aim is to secure the triumph of the truth.

In eulogizing these discoveries, the Anthropological Society has but one object in view, *i. e.*, to render the culture of the truth more fervent, which, aside from the culture of the beautiful and the good, is one of the noblest occupations of that man who desires to see the age of perfection.

